# Investigation of the electromagnetic characteristics and operating performance of a bidirectional PM excited machine

**DOI:** 10.1038/s41598-022-12567-w

**Published:** 2022-05-19

**Authors:** Yanjun Ge, Zhenhan Liu, Kaikai Zhou, Junyue Yang, Dongning Liu

**Affiliations:** 1 School of Mechanical Engineering, Dalian Jiaotong University, Dalian, China; 2 School of Energy and Intelligence Engineering, Henan University of Animal Husbandry and Economy, Zhengzhou, China

**Keywords:** Electrical and electronic engineering, Engineering

## Abstract

This paper presents a study of bidirectional permanent magnet excited machine (BPMEM) based on the study of field-modulation permanent magnetic gear machine (FPGM). The BPMEM structure includes the installation of consequent-pole permanent magnets (PMs) on both the stator and rotor sides of the FPGM so that the stator and rotor can be bidirectionally excited to increase the working airgap flux density amplitude, reduce the flux leakage between poles, and increase the torque density. Therefore, the paper first analysis the influence of different airgap structures and PM arrangements on the airgap flux density and studies the winding slot–pole combination and the resulting working flux density harmonics to analyse the electromagnetic torque generation mechanism. By using the finite element analysis (FEA), the quantitative analysis and comparison of the FPGM, slot-wedge-less FPGM (SWL-FPGM), consequent-pole FPGM (CP-FPGM) and BPMEM verify the superiority of BPMEM in improving electromagnetic torque. In addition, the paper also studies the key performance of BPMEM’s overload capacity, power factor and flux-weakening capability. Finally, no-load and independent load experiments are carried out on the FPGM prototype to verify the correctness of the FEA model and analysis method of the machine in this paper.

## Introduction

The main disadvantages of the existing “asynchronous motor + mechanical gearbox” transmission mode are low transmission efficiency, great wear, poor environmental friendliness, and high maintenance cost. To solve the above problems, the transmission mode of a permanent magnet (PM) motor + PM gear can be adopted.

In 2001, Atallah proposed a field modulated PM gear (FMPMG) transmission model^1^, which can realize frictionless, wear-free, low-speed, high-torque transmission through magnetic field coupling. Applying the FMPMG operating mechanism to existing PM machines, many new composite PM machines can be formed^2–4^, such as magnetically geared machines^5^, ‘pseudo’ direct-drive machines^6^, PM Vernier machines^7–9^, flux reversal PM machines^10,11^ and switched flux PM machines^12,13^. References^14,15^ reveal the modulation mechanism and internal connection of the abovementioned machines with different structures.

Under the premise of further understanding the magnetic field modulation theory, a variety of new magnetic field modulation PM machines have been proposed to further improve the machines’ torque density. Reference^16^ presents a novel magnetic-geared permanent magnet machine with dual-layer PM excitations, and its torque density can reach 79.2 kNm/m^3^. References^17,18^ use dual stator drives to increase the machine airgap area, and the torque density can separately reach 95 kNm/m^3^ and 116 kNm/m^3^. However, the stator and the intermediate ferromagnetic poles (FMPs) of this type of machine have a separate structure, which increases the manufacturing cost and process difficulty and reduces the system reliability.

The recent Dual PM machine usually obtains the performance of the instrument by parameter optimization^19^ or changing the PM structure^20,21^. In addition, Dual PM structure can be used for split tooth PM Vernier machine^22^, hybrid excitation machine^23^ and stator PM machine^24^. Reference^25^ summarizes the contribution of airgap magnetic field density harmonics to no-load back EMF of Dual PM machine. Reference^26^ proposed four Dual-PM Excited Machines, and their magnetic field harmonics and operation principles are theoretically studied.

To solve the above problems, Ref.^27^ proposed a field modulated PM gear machine (FPGM). Its operating principle and electromagnetic torque were studied, its prototypes were trial-produced, experiments were carried out on its load-bearing capacity under different loads. However, there is no experimental analysis of no-load back electromotive force (EMF). This structure also has the disadvantages of large magnetic flux leakage and low efficiency, which limits the further improvement of torque density.

Therefore, based on FPGM, this paper quantitatively analysis the influence of FPGM structure change on machine performance, focuses on the analysis of the relationship between each air gap flux density and electromagnetic torque, the overload and magnetic flux weakening performance of BPMEM. Compares and verifies their electromagnetic performance through finite element analysis. From this, it is concluded that BPMEM torque density is better than FPGM. In the experiment, this paper focuses on the analysis of FPGM no-load back EMF and independent load power generation experiment.

In this paper, one side of the PM of the dual convex airgap structure is equivalent to a smooth surface to establish the equivalent airgap permeance model, further operational mechanism of electromagnetic torque is introduced in “Configuration and operating principle” section. In “Electromagnetic analysis” section, the airgap flux density and electromagnetic torque of different airgap structures are quantitatively compared by using FEA, and the operation performance of BPMEM is analysed. The FPGM prototype is tested, and the no-load back EMF and independent load power generation experimental results which was not tested in reference^27^ are carried out in “Experimental” section. Finally, conclusions are drawn in “Conclusion” section.

## Configuration and operating principle

### No-load airgap magnetic field analysis

**Figure 1:**
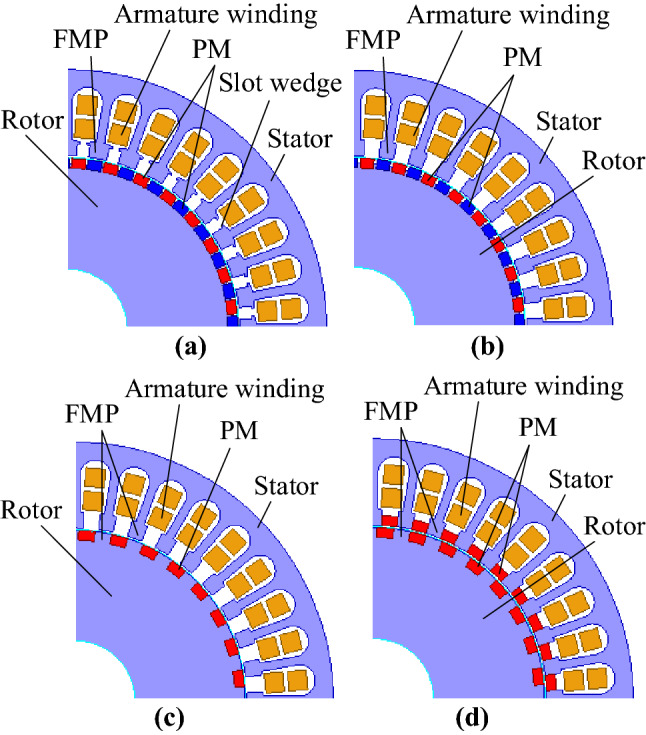
Structure of four kinds of magnetic field modulation PM machines. (**a**) FPGM, (**b**) SWL-FPGM, (**c**) CP-FPGM, and (**d**) BPMEM.

Figure 1 shows the basic electromechanical structure of four kinds of magnetic field modulation PM machines under different airgap structures. Figure 1a shows the FPGM structure based on the “magnetic gear effect”, on the basis of the conventional PM machine to increase the FMP to match the armature winding and rotor PM pole pairs. Figure 1b shows that the slot-wedge-less FPGM (SWL-FPGM) structure of the airgap permeance model is improved by removing the slot wedge structure. Figure 1c is the consequent-pole FPGM (CP-FPGM) structure that only retains the N-pole PMs on the rotor side. Figure 1d shows the BPMEM structure proposed in this paper. The stator is equipped with consequent-pole PMs between the CP-FPGM’s stator FMP, and the rotor has the same structure as the CP-FPGM.

The above four models all have the same winding configuration, number of slots and number of rotor pole pairs.

To simplify the model analysis, the permeance of the steel lamination is infinite, magnetic saturation is not considered, only the modulation harmonic order and speed are considered, and the influence of bias component of consequent-pole PMs’ MMF is ignored. Therefore, only the radial component of the PM magnetomotive force (MMF) can be considered, and the no-load airgap magnetic field tangential component and leakage flux can be ignored. It is also assumed that the permeability of the PM is equal to the permeability of the vacuum.

According to the magnetic circuit method, the PM is equivalent to a constant MMF source in series with the PM reluctance, the airgap is represented by the equivalent airgap specific permeance, and the airgap flux density is the product of the two.

For the CP-FPGM and BPMEM, each pair of poles on the rotor side is composed of unipolar PMs and adjacent FMP. Since the pole arc coefficient of the rotor permanent magnet is 0.5, according to Ref.^28^, the magnetomotive force generated by the permanent magnet is equal to that generated by the adjacent FMP. The stator PM MMF can be analysed in the same way, but the existence of stator slots makes the actual magnetic resistance higher than that of the PM. This paper focuses on the qualitative analysis of the modulation of the stator FMP to the PMs of the rotor and the modulation of the rotor FMP to the PMs of the stator. Therefore, when the consequent poles of the stator and rotor are equivalent to the alternating PM poles, the PM side is equivalent to a smooth surface.

**Figure 2:**
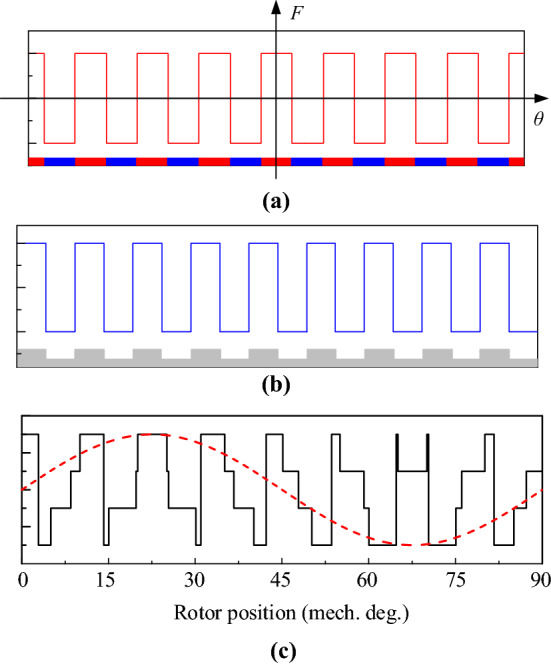
Waveform of MMF, specific permeance and airgap flux density. (**a**) MMF, (**b**) airgap specific permeance, and (**c**) airgap flux density.

Figure 2 shows the equivalent MMF source of the PM and the equivalent airgap model under the action of the FMP, including 16-pole PMs and 9 FMPs. Figure 2a shows the MMF waveform of alternately arranged bipolar PMs, and its amplitude is related to the shape and material of the PM. Figure 2b is a simplified equivalent cogging structure, and the airgap ratio permeability waveform is obtained from this, and its AC variation characteristic reflects the modulation effect of FMP on the MMF. Figure 2c shows the airgap flux density after the airgap MMF is modulated by the airgap specific permeance. The 16-pole MMF can produce 2 poles after modulation by the airgap specific permeance.

The equivalent square wave MMF *F*_*r*_ (*θ, t*) of the rotor PM can be obtained after Fourier decomposition:

1 $$F_{r} (\theta ,t) = \sum\limits_{i = 1,2,3 \ldots } {F_{i} } \cos \left( {ip_{r} \left( {\theta - \omega_{r} t} \right)} \right),$$

where *F*_*i*_ is the amplitude of each harmonic of *F*_*r*_ (*θ, t*), *p*_*r*_ is the number of pole pairs of the PM of the rotor, *ω*_*r*_ is the mechanical angular speed of the rotor, and *F*_*i*_ is calculated as:

2 $$F_{i} = 4B_{r} g_{pm} {\text{sin(}}i\pi {/2)/}i\pi \mu_{{0}} ,$$

where *B*_*r*_ is the remanence of the PM, *g*_*pm*_ is the thickness of the PM, and *μ*_0_ is the vacuum permeability.

The airgap specific permeance distribution affected by the stator FMP is *P*(*θ*), then:

3 $$P(\theta ) = \sum\limits_{j = 0,1,2,3} {P_{j} } \cos \left( {jN_{s} \theta } \right),$$

where the value of *P*_*j*_ is related to the structural size of the slotted side of the airgap and *N*_*s*_ is the number of stator teeth^29^.

Suppose the airgap flux density caused by the rotor PM and stator FMP is *B*_*gr*_ (*θ, t*), then:

4 $$\begin{aligned} B_{gr} (\theta ,t) &= F_{r} (\theta ,t) \cdot P(\theta ) \\ &= \frac{1}{2}\sum\limits_{i = 1,2,3 \ldots j = 0,1,3 \ldots } {P_{j} } F_{i} \cos \left( {\left( {ip_{r} \pm jN_{s} } \right)\left( {\theta - \frac{{ip_{r} \omega_{r} }}{{ip_{r} \pm jN_{s} }}t} \right)} \right). \\ \end{aligned}$$

The airgap flux density caused by the rotor consequent-pole PM and stator FMP is *B*_*gr*_*ˊ* (*θ, t*) = *B*_*gr*_ (*θ, t*) + *B*_*p*_, where *B*_*p*_ is the airgap magnetic field bias caused by rotor PM modulated by rotor FPM. Because the modulation does not introduce new harmonics and only changes the original magnetic density amplitude^30^, it is can be expressed by the bias coefficient.

The equivalent square wave MMF of the stator PM can be expressed as:

5 $$F_{s} (\theta ,t) = \sum\limits_{m = 1,2,3 \ldots } {F_{m} } \cos \left( {ip_{s} \theta } \right),$$

where *p*_*s*_ is the number of pole pairs of the stator PMs, and *F*_*m*_ adopts equivalent PM thickness. Therefore, the contribution of the stator PMs to the airgap flux density is usually less than that of the rotor PMs.

The airgap specific permeance distribution affected by the rotor FMP is *P*_*r*_ (*θ*), then:

6 $$P_{r} (\theta ) = \sum\limits_{n = 0,1,2,3} {P_{rn} } \cos \left( {nN_{r} \left( {\theta - \omega_{r} t} \right)} \right),$$

where *N*_*r*_ is the number of rotor teeth.

Set the airgap flux density caused by the stator consequent-pole PM and rotor FMP as *B*_*gs*_ (*θ, t*), then:

7 $$\begin{aligned} B_{gs} (\theta ,t) &= F_{s} (\theta ,t) \cdot P_{r} (\theta ) + B_{p} \\ &= \frac{1}{2}\sum\limits_{m = 1,2,3 \ldots n = 0,1,3 \ldots } {P_{n} } F_{m} \cos \left( {\left( {nN_{r} \pm mp_{s} } \right)\left( {\theta - \frac{{nN_{r} \omega_{r} }}{{nN_{r} \pm mp_{s} }}t} \right)} \right) + B_{p} . \\ \end{aligned}$$

Equations (4) and (7) show that the PMs in the machine can be modulated to produce multiple airgap flux densities with different rotor speeds, as shown in Table 1.

**Table 1:** Airgap flux density harmonics under a no-load condition.

	Pole pairs	Angular speed	Frequency
Rotor PM	$\left| {ip_{r} \pm jN_{s} } \right|$	$\frac{{ip_{r} \omega_{r} }}{{ip_{r} \pm jN_{s} }}$	$\frac{{ip_{r} \omega_{r} }}{2\pi }$
Stator PM	$\left| {nN_{r} \pm mp_{s} } \right|$	$\frac{{nN_{r} \omega_{r} }}{{nN_{r} \pm mp_{s} }}$	$\frac{{nN_{r} \omega_{r} }}{2\pi }$

Table 1 shows that due to *p*_*r*_ = *N*_*r*_, when *i* = 1 or *n* = 1, the airgap flux density harmonics have the same frequency. At this time, the effective airgap flux density harmonic orders generated by the stator and rotor PMs include *|p*_*r*_ ± *jN*_*s*_| and *|N*_*r*_ ± *mp*_*s*_|.

To simplify the analysis, only the *p*_*r*_, *|p*_*r*_ − *N*_*s*_|, and *|N*_*r*_ − *p*_*s*_| harmonics in the airgap magnetic field are considered, and the order and speed of the latter two are the same.

### Armature winding analysis

The electromotive force and magnetomotive force of each phase in the three-phase winding shall be symmetrical, so the stator winding of the BPMEM is designed as 36-slot 8-pole. According to the number of armature winding pole pairs *p*_*a*_ satisfies *p*_*a*_ = *N*_*r*_ − *p*_*r*_, the maximum output torque can be obtained^31^, the number of rotor poles is designed as 64-pole, and their pitch and winding factor are 4 and 0.945, respectively.

Table 2 shows the specific structural parameters of the four models shown in Fig. 1.

**Table 2:** Main parameters of four kinds of magnetic field modulation PM machines.

	FPGM	SWL-FPGM	CP-FPGM	BPMEM
Stator outer diameter, mm	210			
Stack length, mm	105			
Airgap length, mm	1			
Rotor outer diameter, mm	138			
Number of winding poles	8			
Winding type	Double layer			
Number of rotor poles	64			
Phase	3			
Number of slots	36			
Stator FMP duty ratio	0.6	0.5	0.5	0.5
Stator PM thickness, mm	–	–	–	4
Rotor PM thickness, mm	4			
Rotor PM width, mm	5.4	5.4	6.7	6.7
Turns in series per phase	502			
Magnet remanence, T	1.12			
Slot area, mm^2^	239.8			
slot filling factor	73%			

**Figure 3:**
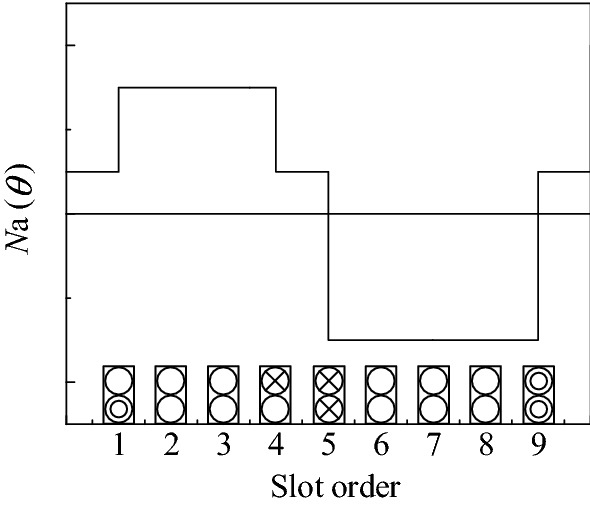
A-phase winding function.

Figure 3 shows the A-phase winding function *N*_*a*_ (*θ*) of the BPMEM. Table 2 shows that the windings of these four models are three-phase symmetrical. In combination with Fig. 3, the positive and negative half-axis components of the winding function of each phase are asymmetric, which produces even-order MMF harmonics.

When the three-phase winding shown in Fig. 3 is supplied with a sinusoidal alternating current, it generates a *p*_*a*_ fundamental armature MMF and a counter-rotating *N*_*s*_ − *p*_*a*_ harmonics armature MMF. The latter is tooth harmonics in the usual sense, which is the eighth harmonic relative to the fundamental magnetomotive force of the winding, and its winding factor is the same as the fundamental wave, its amplitude is 1/8 of the fundamental wave.

**Figure 4:**
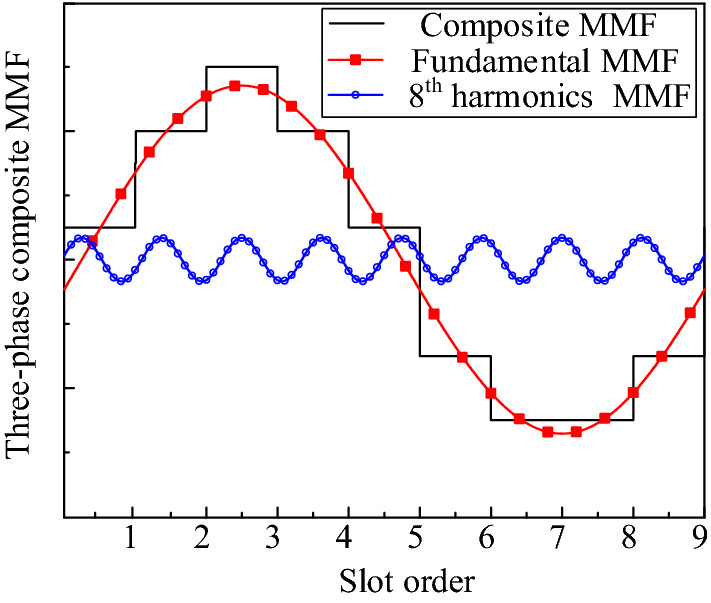
Three-phase composite MMF.

Figure 4 shows the three-phase composite MMF at a certain moment when a direct current of *I*_*b*_ = *I*_*c*_ =  *− I*_*a*_*/*2 is applied to each model winding, as shown in Fig. 1. It is found that when the above four structures are connected with three-phase AC, the *p*_*r*_th three-phase synthetic magnetomotive force tooth harmonic generated by the winding is equal to the number of fundamental magnetomotive force poles of the rotor PMs and rotates synchronously. The (*N*_*s*_ − *p*_*r*_)th harmonic generated by the modulation of the stator and rotor PMs is equal to the pole number of winding fundamental magnetomotive force and rotates synchronously. Therefore, both *p*_*r*_th and (*N*_*s*_ − *p*_*r*_)th harmonics in the air gap are working harmonics, which indicates that there are multiple harmonics in the magnetic field modulation PM machines shown in Fig. 1, and the electromagnetic torque is generated by these multiple harmonics. According to the principle of traditional permanent magnet synchronous motor, two magnetic fields with the same number of poles produce stable torque when the speeds of two magnetic fields are synchronized.

## Electromagnetic analysis

### Torque analysis

The three-phase winding has symmetry, so only one of the phases need to be analysed. According to winding function theory^32^, the no-load flux linkage of the phase A winding can be expressed as:

8 $$\psi_{a} = r_{g} L_{stk \, } \int_{0}^{2\pi } {B_{g} } \left( {\theta ,t} \right)N_{a} \left( \theta \right)d\theta ,$$

where *r*_*g*_ is the airgap radius, *L*_*stk*_ is the stack length, *B*_*g*_ = *B*_*gr*_ + *B*_*gs*_, and only BPMEM exists *B*_*gs*_.

Equation (8) shows that the winding has a filtering effect on the airgap flux density; that is, the non-existent harmonic of the winding MMF cannot induce the corresponding back-EMF. In this paper, the *p*_*a*_th and *p*_*r*_th flux densities in the airgap produce the same frequency back-EMF in the winding, which contributes to the electromagnetic torque, which is consistent with the analysis in “Armature winding analysis” section.

According to AC machine dynamic analysis theory and the Ref.^6^, when *I*_*d*_ = 0, the electromagnetic torque can be expressed as:

9 $$T_{e} = \frac{3}{2}p_{r} \psi_{d} I_{q} = 3kNr_{g} l_{stk} I_{q} \left( {B_{gpr} + \frac{{p_{r} }}{{p_{a} }}B_{gpa} } \right),$$

where *ψ*_*d*_ is the value in the *d*-axis direction of the flux linkage after *d-q* transformation, *N* is turns in series per phase, *B*_*gpr*_ is the amplitude of the *p*_*r*_th airgap flux density by the production of PM MMF and specific permeance, and *B*_*gpa*_ is the amplitude of the *p*_*a*_th airgap flux density by the production of PM MMF and the specific permeance.

Equation (9) shows that when the specific electric loading is constant, the average electromagnetic torque of the machine can be increased by increasing the amplitude of the no-load airgap flux density. The contribution of the *p*_*a*_th airgap flux density to torque is greater than that of the *p*_*r*_th airgap flux density. The SWL-FPGM is obtained by changing the structure of the stator FMP on the basis of the FPGM by changing the influence of *P*_0_ and *P*_1_ on the airgap flux density, appropriately reducing the duty ratio of the stator FMP to reduce the *P*_0_ amplitude, increasing the *P*_1_ amplitude, and then increasing the *B*_*gpa*_ amplitude. For the CP-FPGM, the thickness of the equivalent PM is reduced, which can reduce the equivalent airgap reluctance. In actual situations, however, the presence of rotor FMP make the air gap permeance distribution change with rotor rotation, resulting in larger torque ripples^29^. In the BPMEM structure, PMs are installed on the stator side, which are modulated by rotor FMPs to obtain the *p*_*a*_th airgap flux density, thereby increasing the electromagnetic torque.

**Figure 5:**
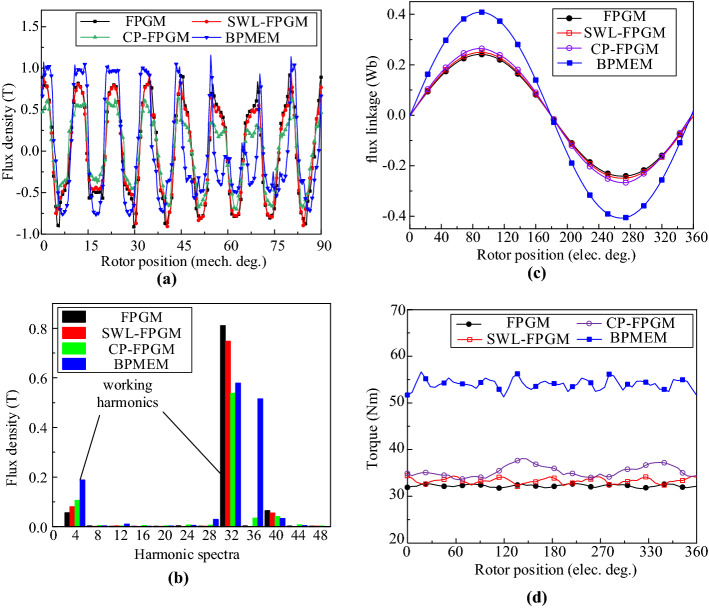
Electromagnetic characteristics. (**a**) No-load airgap flux density waveform, (**b**) no-load airgap flux density harmonic spectrum, (**c**) no-load flux linkage of the A-phase winding, and (**d**) electromagnetic torque (5 A/mm^2^).

According to the structural parameters shown in Table 2, the electromagnetic characteristics of the four models shown in Fig. 5 can be obtained through FEA. Figure 5a is the no-load (only PM generates a magnetic field) airgap flux density waveform. Figure 5b is the no-load airgap flux density harmonic spectrum. Figure 5c is the no-load flux linkage of the A-phase winding generated by the machine rotor rotating at an electrical angle of 360° and Fig. 5d is the electromagnetic torque generated when the current density is 5 A/mm^2^ under the control of *I*_*d*_ = 0.

Figure 5a,b show that different machine structures have different effects on the no-load airgap flux density. Therefore, when only PMs generate magnetic fields, the FPGM has the highest 32nd airgap flux density amplitude, where 32 is the same as the number of pole pairs of rotor PMs. The SWL-FPGM and CP-FPGM have a higher 4th airgap flux density amplitude, where 4 is the same as the number of pole pairs in the armature winding, indicating that the latter two have a better modulation effect, while BPMEM benefits from PM bidirectional modulation, so that its 4th working airgap flux density amplitude is greatly increased. In addition, the 36th harmonic of BPMEM is mainly produced by stator PMs, which are stationary relative to the stator and do not participate in the energy conversion of the machine.

From Fig. 5b, it can also be seen that the 32nd harmonic can be improved by stator PM. The causes can be explained by formula (7) and Table 1, when *m* and *n* are both 4, the magnetic field generated by the stator PM can be modulated to 32nd harmonic, so it can improve the 32nd harmonic. This also proves that BPMEM has the same dual flux modulation effect as reference^33^.

Figure 5c shows that the amplitude of the no-load flux linkage of the phase A winding of BPMEM is the largest, followed by the CP-FPGM, again without the SWL-FPGM, and that of the FPGM is the smallest. Equation (8) shows that this is because the no-load flux linkage is greatly affected by the 4th working harmonic that matches the number of pole pairs of the armature winding.

Figure 5d shows that when the input current density is constant, the average electromagnetic torque increases with increasing no-load flux linkage. BPMEM has the largest average electromagnetic torque, which is 54 Nm (34.4 kNm/m^3^), the second is the CP-FPGM, which is 35.4 Nm (22.5 kNm/m^3^), the third is the FPGM without a slot wedge, which is 33.3 Nm (21.2 kNm/m^3^) and the FPGM is the smallest, with a value of 32 Nm (20.4 kNm/m^3^). The average electromagnetic torque of the BPMEM is improved by approximately 68% compared with the FPGM, and the relationship between the average electromagnetic torque and the no-load flux linkage is in accordance with the description of Eq. (9).

### BPMEM operating performance analysis

Limited by the insulation withstand voltage of the power supply equipment and the machine, under the premise of the same slot area and slot filling factor, turns in series per phase of BPMEM are redesigned to ensure that the machine specific electric loading remains unchanged. The output performance of the torque, copper loss, iron loss and power factor are not affected before and after the change. With 300 rpm as the turning speed, the number of turns in series per phase of BPMEM is selected as 276.

**Figure 6:**
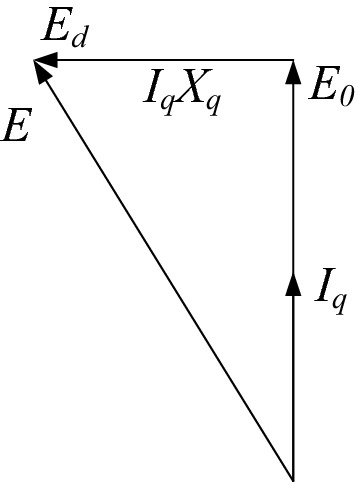
Phasor diagram.

Figure 6 is a phasor diagram of a PM machine under *I*_*d*_ = 0 control when the motor convention is adopted. Here *E* is the back-EMF generated by the change in the flux linkage, *E*_0_ is the no-load back-EMF, *X*_*q*_ is the *q*-axis reactance, *ω* is the electrical angular speed, *ψ* is the airgap composite flux linkage, *L*_*q*_ is the *q*-axis inductance, and in mathematical terms, *E* = *ωψ* and *X*_*q*_ = *ωL*_*q*_.

Ignoring the stator winding resistance and end leakage reactance, the power factor of the machine can be obtained from Fig. 6 as:

10 $$\cos \varphi = \frac{1}{{\sqrt {1 + \left( {\frac{{I_{q} L_{q} }}{{\psi_{d} }}} \right)^{2} } }}.$$

Equation (10) shows that when *I*_*d*_ = 0, the power factor is mainly affected by *L*_*q*_ and the *d*-axis flux linkage *ψ*_*d*_. The influence of the *d-*axis inductance *L*_*d*_ should also be considered when the machine is operated in flux-weakening and flux-enhancing conditions. When the magnetic saturation of the machine is taken into account, *L*_*dq*_ and *ψ*_*d*_ vary with changes in load and operating conditions.

**Figure 7:**
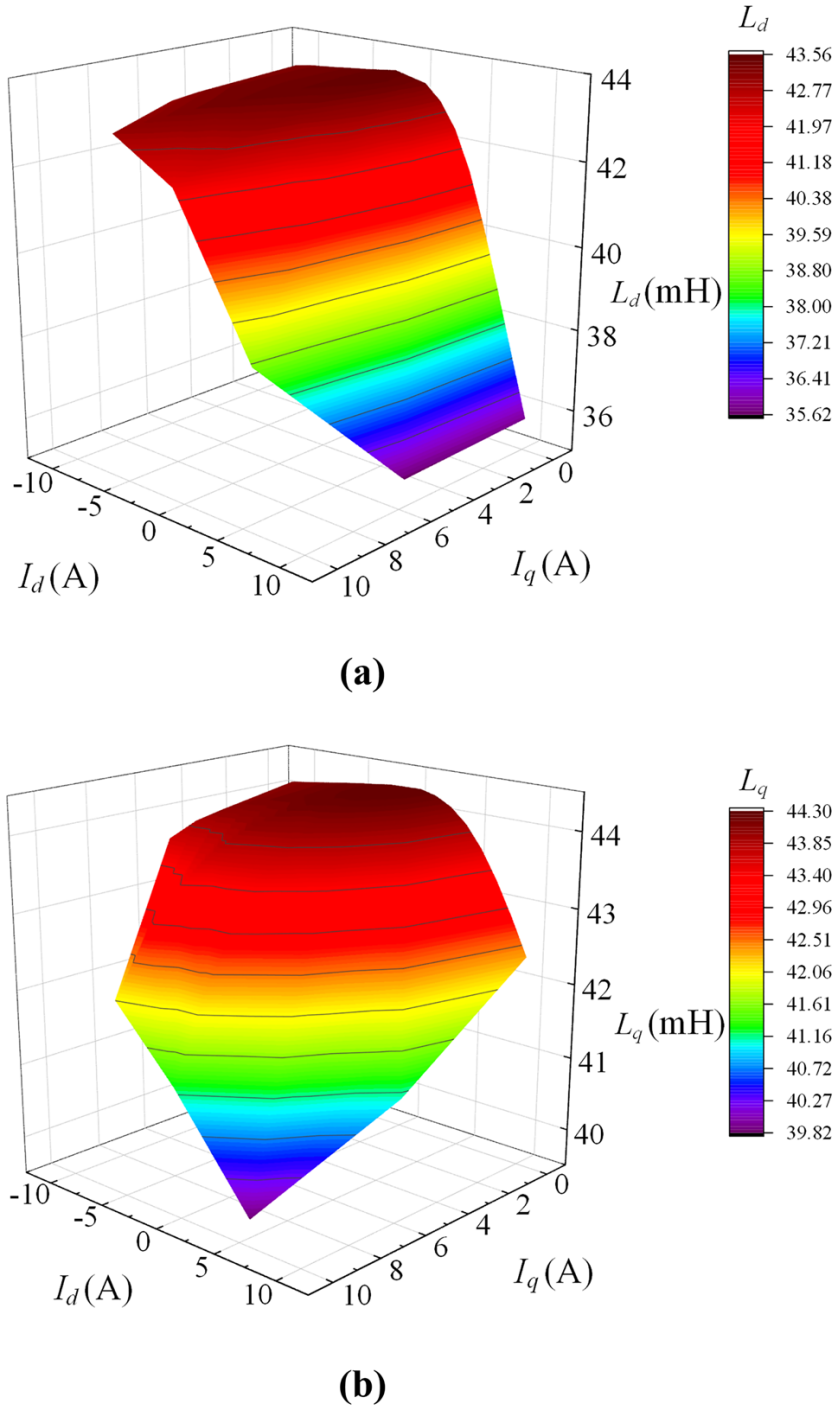
Influence of *I*_*d*_ and *I*_*q*_ on *L*_*d*_ and *L*_*q*_. (**a**) Influence of *I*_*d*_ and *I*_*q*_ on *L*_*d*_, (**b**) influence of *I*_*d*_ and *I*_*q*_ on *L*_*q*_.

Figure 7 shows the relationship between the current amplitude and *L*_*dq*_ in different phases. Due to the symmetrical structure of the machine, *L*_*dq*_ is symmetrical with respect to *I*_*q*_ = 0. Here, only the BPMEM in the electric state is analysed.

As seen from Fig. 7a, the change in *L*_*d*_ mainly comes from the influence of *I*_*d*_, and the change in *L*_*d*_ at flux weakening is much smaller than the change in *L*_*d*_ at the operating condition of flux-enhancing because the state of flux-enhancing can cause the iron core to enter magnetic saturation, which in turn reduces the magnetic conductivity of the iron core.

As shown in Fig. 7b, the change in *L*_*q*_ mainly comes from the influence of *I*_*q*_. Due to the coupling between the *q*-axis magnetic circuit and the *d*-axis magnetic circuit in the iron core, the PM and *I*_*d*_ also have a significant impact on *L*_*q*_.

**Figure 8:**
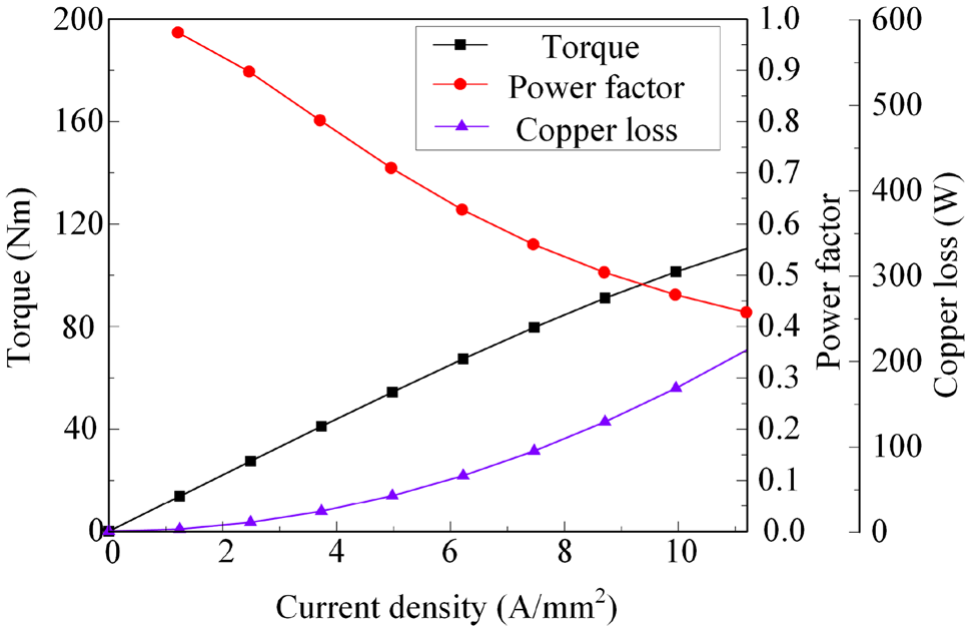
Electromagnetic torque, power factor, copper loss vs current density.

**Figure 9:**
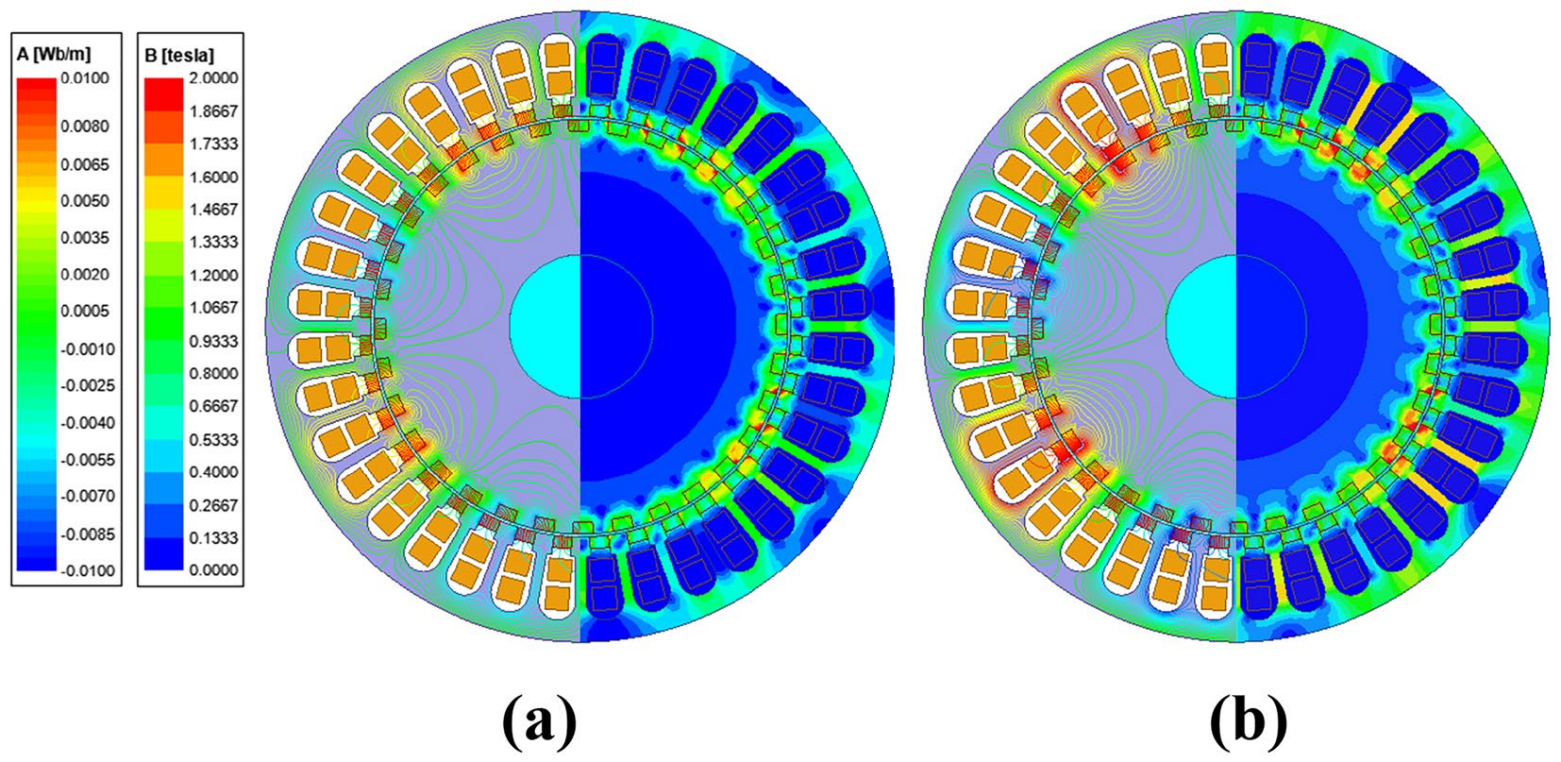
Flux density cloud map and flux line distribution. (**a**) 5 A/mm^2^, (**b**) 10 A/mm^2^.

Figure 8 shows the relationship curve of electromagnetic torque, power factor and copper loss vs current density when *I*_*d*_ = 0. Figure 9 corresponds to Fig. 8 when the current density is 5 A/mm^2^ and 10 A/mm^2^, respectively, representing the flux density cloud map and the flux line distribution.

It can be seen from the torque curve shown in Fig. 8 that the electromagnetic torque of the BPMEM is positively related to the current density. Affected by the iron core B–H curve, the electromagnetic torque rises slowly when the current density is approximately 8 A/mm^2^. In combination with Fig. 9, it can be observed that when the current density is 10 A/mm^2^, the teeth show obvious magnetic saturation compared to the situation for 5 A/mm^2^.

The power factor curve shown in Fig. 8 and Eq. (10), show that the power factor of the machine decreases with increasing current density, and the copper loss is proportional to the square of the current density. Considering the temperature rise of the machine, the current density was 5 A/mm^2^ as the rated state. At this time, the corresponding machine output torque is 54 Nm, the copper loss is 42 W, and the power factor is 0.707.

**Figure 10:**
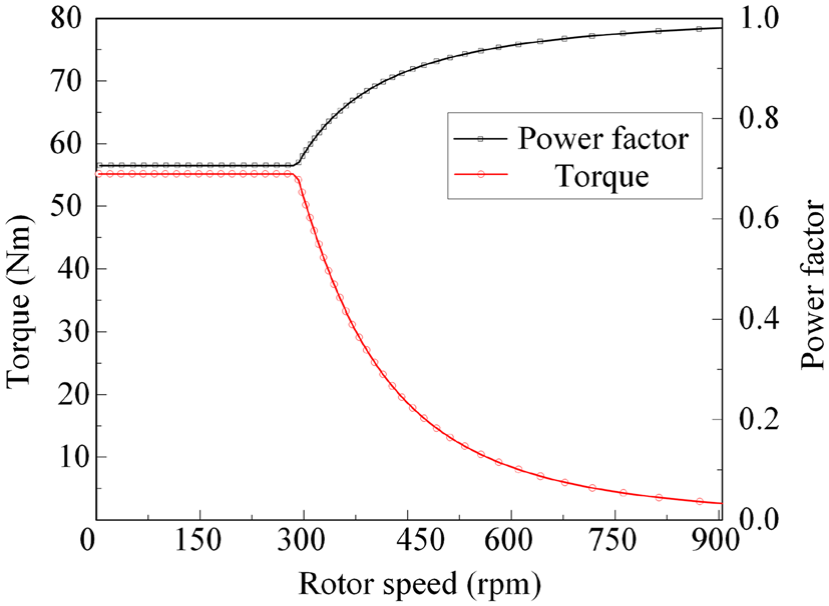
Power factor and torque under the flux-weakening condition.

Figure 10 shows the power factor and torque characteristic curves of BPMEM under flux-weakening conditions.

It can be seen from Fig. 10 that when the speed is below the fundamental frequency (the corresponding speed is 300 rpm), BPMEM runs in the constant torque region, and the power factor is also a constant value at this time, when the speed exceeds 300 rpm, BPMEM enters the flux-weakening control and runs in the constant power region, and the torque decreases with increasing speed. However, due to the change in the phase difference between the current and the voltage, the power factor increases instead.

When the iron core is placed in an alternating magnetic field, due to the existence of magnetic domain friction and eddy current, the machine iron core produces losses.

Suppose the core loss of BPMEM is $p_{Fe}$, then there is^34^

11 $$p_{Fe} = p_{h} + p_{c} = k_{h} fB_{m}^{2} + k_{e} f^{2} B_{m}^{2} ,$$

where $p_{Fe}$ is the hysteresis loss, is the eddy-current loss, *f* is the alternating frequency of the magnetic field, $B_{m}$ is the flux density amplitude, and $k_{h}$ and $k_{e}$ are the hysteresis loss and the eddy-current loss coefficients, respectively, which are related to the core material, volume, and laminate thickness. In the FEA, D23_50 silicon lamination is adopted, and *k*_*h*_ and *k*_*e*_ are set to 325.26 and 0.865 respectively. In addition, the excess losses caused by processing and manufacturing are not included.

**Figure 11:**
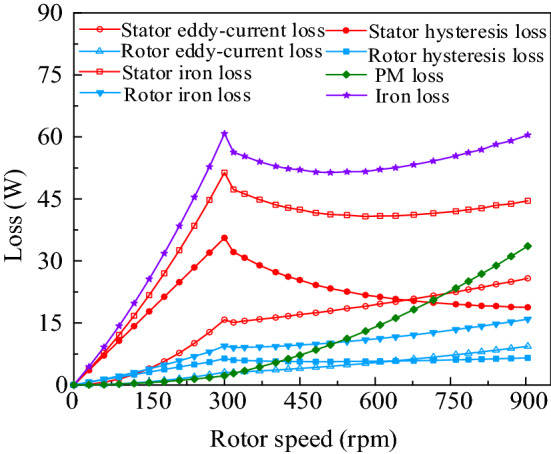
Iron loss and PM loss of BPMEM under the flux-weakening condition.

Figure 11 shows the iron loss and PM loss of BPMEM at different speeds.

Figure 11 shows that the stator and rotor hysteresis losses increase linearly with increasing speed, and the stator and rotor eddy-current losses are approximately quadratic with increasing speed, the PM loss mainly manifests as eddy-current loss, which is also positively correlated with the square of the speed. The stator iron loss is significantly higher than the rotor iron loss because the rotor rotates synchronously with the fundamental working magnetic field, and the relative frequency between the rotor and magnetic field is zero. It can also be seen from Fig. 11 that when the BPMEM is running at a speed of 300 rpm or more, due to the flux weakening of the armature current, the stator and rotor iron hysteresis loss is reduced, and the rising trend of the iron eddy-current loss also tends to slow down.

In summary, when BPMEM runs at 300 rpm, its copper loss, iron loss, and PM loss are 42 W, 60.8 W, and 2.2 W, respectively. If mechanical friction, air resistance and other stray losses are not considered, the rated operating efficiency of BPMEM is 93.9%. Compared with fractional slot concentrated winding permanent magnet synchronous motor, which is also used in low-speed and high torque occasions, BPMEM adopts double excitation structure and introduces a large number of magnetic field harmonics, which increases the iron consumption. However, it is the increase of magnetic field harmonics that makes the electromagnetic torque of bpmem much higher than that of fractional slot concentrated winding permanent magnet synchronous motor under the same size and current input conditions. Thus, the torque density of the motor is increased.

## Experimental

**Figure 12:**
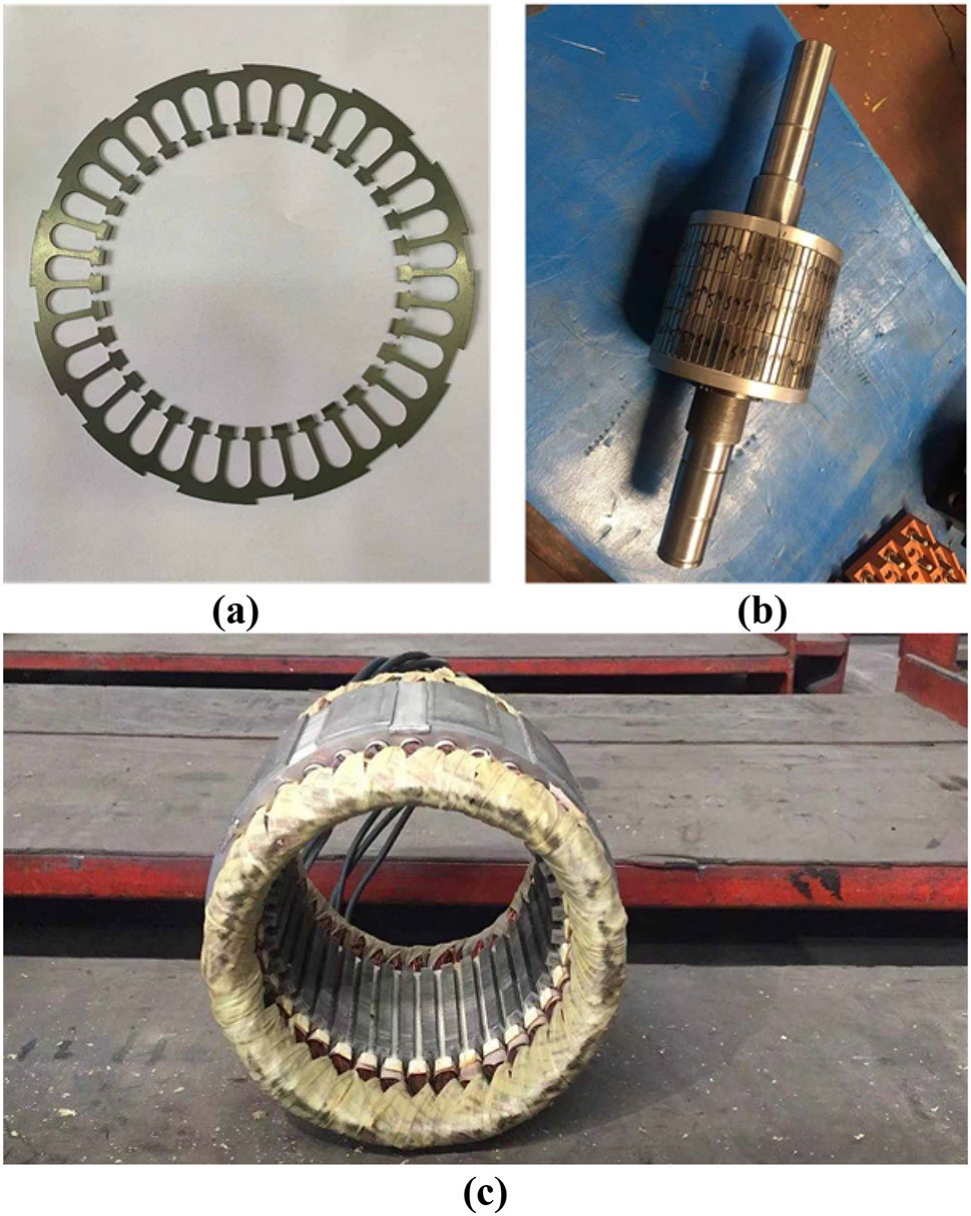
Photos of the FPGM prototype machine. (**a**) Stator steel lamination sheet, (**b**) rotor, (**c**) stator.

In order to further test the performance of FPGM and provide better guidance for the prototype trial production of BPMEM, this paper further tests the performance of FPGM on the basis of Ref.^19^. The experimental prototype of FPGM is shown in Fig. 12.

Figure 12a shows an FPGM steel lamination sheet, Fig. 12b shows an FPGM rotor, and Fig. 12c shows an FPGM stator, and were taken by Junyue Yang.

**Figure 13:**
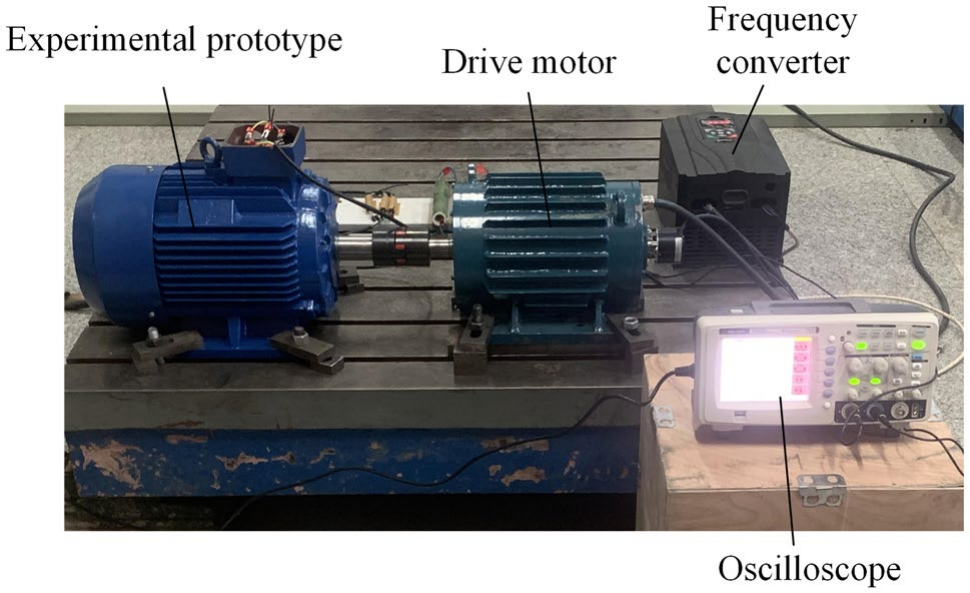
FPGM prototype experimental platform.

Figure 13 shows a FPGM prototype experimental platform and was taken by Junyue Yang. In Fig. 13, the drive motor drags the FPGM prototype to rotate to produce the corresponding back-EMF. No-load and independent load experiments are carried out on the FPGM prototype, and the results are compared with the FEA results of the corresponding model.

**Figure 14:**
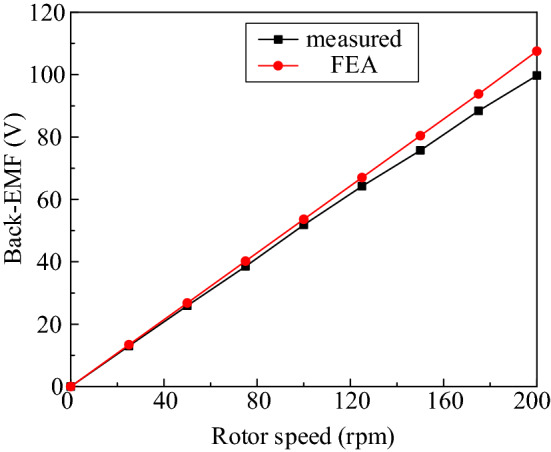
Comparison of the measured and FEA effective values of no-load back-EMF at different speeds.

Figure 14 shows the no-load back-EMF curve of the FPGM by FEA and experimental measurement.

In Fig. 14, the FEA of the no-load back-EMF is basically consistent with the measured value curve and increases with increasing rotor speed. Due to the neglection of excess loss and post manufacturing loss i.e. (laser cutting, punching), there is an overestimation of the simulated back-EMF w.r.t. the measured value, and this mismatch is getting bigger at higher speeds.

**Figure 15:**
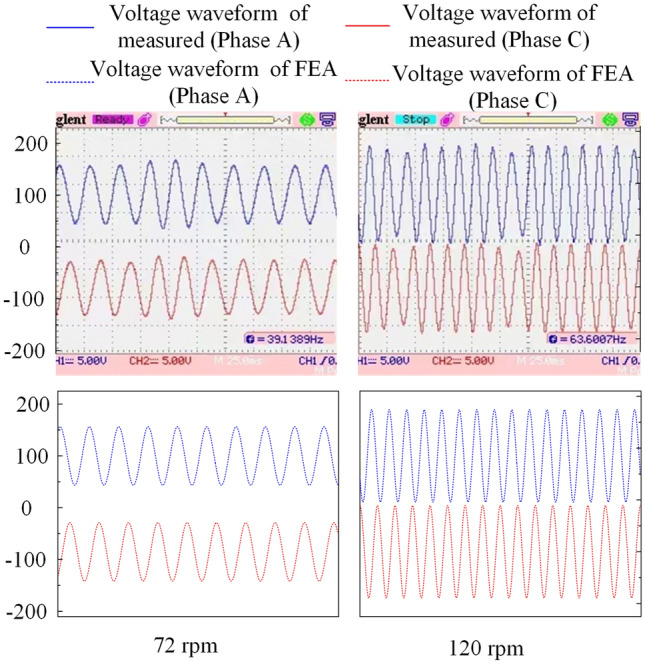
Comparison of measured and FEA voltage waveforms.

Figure 15 shows the measured and FEA voltage waveforms of the FPGM when running under an independent load power generation. The upper half of Fig. 15 shows the load end voltage waveforms of the phase A (upper side) and phase C (lower side) measured by an oscilloscope when the three-phase winding of the FPGM is connected to a 100 Ω resistive load at 72 rpm and 120 rpm, respectively, where the oscilloscope represents a 5 V vertical change per grid, 25 ms horizontally per grid, the oscilloscope probe attenuation coefficient is set to 10x. The lower half of Fig. 15 simulates the waveform by FEA at the corresponding given speed. As seen from Fig. 15, at different speed, the frequencies of the measured and FEA voltage at the load end are basically the same, at 38.4 Hz and 64 Hz, respectively.

Comparing the upper and lower parts of Fig. 15, it can be seen that the measured voltage waveform at different speeds fluctuates in the amplitude domain compared with the finite element analysis, which is caused by the fluctuation of load and air gap magnetic density. On the whole, there is little difference between the measured value and the finite element value in amplitude and frequency domain, which verifies the rationality of the FEA method.

Table 3 shows the measured and FEA effective value of voltage when running under an independent load power generation. The three-phase winding of the FPGM is also connected to a 100 resistive load.

**Table 3:** Comparison of measured and FEA effective value of voltage.

Speed, rpm	Measured, V	FEA, V
24	12.4	13.2
48	24.5	26.1
72	36.2	38.1
96	46.4	49.1
120	55.7	60.2
144	64	71
168	71.7	78.2
192	78.7	86.1
216	85.1	94.8
240	91.1	99.1

It can be seen from Table 3, at 72 rpm, the voltage effective values of the experimental measurement and FEA are 36.2 V and 38.1 V, respectively. When the speed is 120 rpm, the voltage effective values of the experimental measurement and FEA are 55.7 V and 60.2 V, respectively, and the error values of both sets of data are within 8%. Therefore, if not consider the speed fluctuation of the drive motor, the experimental waveform is basically the same as the FEA waveform, which demonstrates the correctness and reliability of the FEA model built in this paper.

## Conclusion

The theoretical and FEA of magnetic field modulation PM machines of different structures is carried out, and the following conclusions are obtained by measuring the no-load back-EMF of the FPGM at different speeds and the load end voltage when operating under independent load:

1. The electromagnetic torque of the magnetic field modulation PM machine is produced by the armature winding and the stator and rotor PMs under the combined action of multiple harmonic magnetic fields in the airgap and is characterized by the same harmonic order and the same speed.
2. The winding function of 36 slot 8-pole stator winding is decomposed by Fourier transform. It is found that the harmonic number obtained is consistent with the harmonic number calculated by stator winding open circuit. The error of the FPGM FEA and prototype experimental measurement is small, which can ensure the correctness of the FEA.
3. The CP-FPGM and BPMEM form more harmonic magnetic fields in the airgap due to the dual convex airgap structure, resulting in significant torque fluctuations. The direct axis and quadrature axis inductance under different loads and working conditions are analysed to obtain a more accurate control model.
4. The machine performance can be improved by changing the stator and rotor PM structure, position and modulation structure, of which the electromagnetic torque of BPMEM is 68% higher than that of the FPGM.
5. The BPMEM gives full play to the characteristics of low-speed high torque, which has good operating characteristics under different load and speed conditions. The comparative study of the four structures also Laid the foundation for the development of low-speed high torque machine.
6. The experimental results of independent load power generation of FPGM are in good agreement with the FEA results, which verifies the correctness of the FEA. Moreover, the generation voltage is sinusoidal, which provides the possibility for the application of FPGM in different occasions.
